# Patient-Reported Experiences in Accessing Primary Healthcare among Immigrant Population in Canada: A Rapid Literature Review

**DOI:** 10.3390/ijerph17238724

**Published:** 2020-11-24

**Authors:** Bishnu Bahadur Bajgain, Kalpana Thapa Bajgain, Sujan Badal, Fariba Aghajafari, Jeanette Jackson, Maria-Jose Santana

**Affiliations:** 1 Department of Community Health Sciences, Cumming School of Medicine, University of Calgary, Calgary, AB T2N 4N1, Canada; bishnu.bajgain@ucalgary.ca (B.B.B.); fariba.aghajafari@ucalgary.ca (F.A.); 2 AabKa Research & Consultancy, Calgary, AB T1Y 5J2, Canada; k.thapab@aabka.ca; 3 School of Medicine, University of Minnesota, 420 Delaware St SE, Minneapolis, MN 55455, USA; badal014@umn.edu; 4 Department of Family Medicine, Cumming School of Medicine, University of Calgary, Calgary, AB T2N 1N4, Canada; 5 Health Quality Council of Alberta, Calgary, AB T2N 2A4, Canada; jeanette.jackson@hqca.ca

**Keywords:** access barriers, access facilitators, immigrant, patient experiences, primary healthcare, Canada

## Abstract

(1) Background: Immigrants represent around 21.9% of the total population in Canada and encounter multifaceted obstacles in accessing and receiving primary healthcare. This literature review explores patient experiences in primary care from the perspective of immigrants and identifies areas for further research and improvement. (2) Methods: A comprehensive search was performed on PubMed, MEDLINE, Embase, SCOPUS, and Google scholar to identify studies published from 2010 to July 2020. Relevant articles were peer-reviewed, in English language, and reported patient experiences in primary healthcare in Canada. (3) Results: Of the 1566 searched articles, 19 articles were included in this review. Overall, the finding from articles were summarized into four major themes: cultural and linguistic differences; socioeconomic challenges; health system factors; patient–provider relationship. (4) Conclusion: Understanding the gaps to accessing and receiving appropriate healthcare is important to shape policies, enhance the quality of services, and deliver more equitable healthcare services. It is therefore pertinent that primary healthcare providers play an active role in bridging these gaps with strong support from policymakers. Understanding and respecting diversity in culture, language, experiences, and systems is crucial in reducing health inequalities and improving access to quality care in a respectful and responsive manner.

## 1. Introduction

Patient experience comprises a range of interactions with the healthcare system, including interacting with doctors, nurses, therapists, and other healthcare staff and facilities [1]. It is one of the fundamental determinants of healthcare quality, patient well-being, and clinical effectiveness [1,2]. A good patient experience broadly encompasses respect, effective communication, shared decision-making, physical comfort, emotional support, and continuity/timely access to care [3]. Primary Health Care (PHC) is the first point of contact for patients to experience a healthcare system where patients initiate their access to medical care with a general practitioner (GP) or family physician (FP) [4]. Per WHO, the three pillars of PHC—meeting people’s lifelong healthcare needs, addressing broader determinants of health, and empowering patients and communities to take charge of their own health—are the foundations as well as major challenges to any healthcare system. Thus, the right to equal and equitable care is the fundamental premise of PHC. Accordingly, access to primary care services is a right for all individuals and communities. Under Canada’s publicly funded universal healthcare system, PHC is the everyday gateway for the majority of Canadians seeking medical services.

Canada is a multicultural society with diverse ethnocultural landscape shaped by years of immigration. Canada is well-known for its friendly immigration policies and for being a welcoming place for immigrants from all over the world [5]. Immigrants represent over one fifth (21.9%) of the Canadian population, which is an increasing trend [6]. Yearly, Canada receives almost 300,000 immigrants [7], with individual or communal healthcare needs varying across all groups. Health status is varied across the subgroups of immigrations (immigrants, labor migrants, refugee, undocumented migrants) because the health of immigrants’ is a consequence of various sociodemographic factors including economic, environment, social-cultural, healthcare delivery system of their home country before they migrate, as well as the postmigration factors, such as new environment, employment, education, economic status, and accessibility and responsiveness of healthcare system. This is also true that the health status is not equivalent with migrants arriving from different parts of the world, such as Asia, Africa, and Middle East. The access of healthcare, education, social services, and other legal right might be varied across the migrants because of their migration status (e.g., labor migrants undocumented migrants). Generally, immigrants arrive in Canada with similar or better health status than the general Canadian population because of the self-selection process for immigration, i.e., those who choose to migrate are usually healthier and have had higher education [8,9]. However, reports also show that the health condition of immigrants, living in Canada for 10 years or more, is worse than those who recently moved to Canada [5,10]. Various research have shown that several causative elements, including socioeconomic, cultural, environmental, systemic, and other social gradients are associated with this deterioration [11]. Adjusting in a new country can be an extremely stressful process. Moreover, understanding a new healthcare system, particularly, for those who have come from vastly different healthcare structures can be even more challenging. Likewise, economic stability could be the priority for newcomers, which may influence individual healthcare seeking behaviors. Therefore, institutional, cultural, and financial barriers may directly impact overall access to PHC for many new immigrants. Barriers in healthcare are anything that restricts or makes it more difficult for individuals in accessing, using, or benefitting from healthcare services. On the other hand, facilitators are factors that support access and continuity of care. Understanding social determinants of health, (such as income and social status/support; education and health literacy; employment/working condition; social and physical environment; personal health practices, believe, culture, and coping strategies), barriers in accessing healthcare, and facilitating factors are important to care providers and healthcare systems to reduce/overcome these barriers and improving the access of care and quality of care [12].

Worldwide, policymakers are steadily interested in gathering and analyzing patient experience data to assess these barriers and determinants in an effort to improve and promote quality of care for all individuals [2].

Evidence shows that newcomers to Canada report challenges in accessing and receiving PHC. Known challenges and facilitators include factors such as culture, language, societal influence, politics, gender expectations, communication, cost, schedule, and the current structure of the healthcare system [5,13]. Adequate access to quality care in PHC is crucial to everyone, but it is still unclear if this goal is being achieved. This rapid literature review is intended to explore patient experiences with PHC from the perspective of immigrant populations in Canada and identify areas for further research and improvement.

## 2. Materials and Methods

### 2.1. Search Strategy and Study Selection

Study selection and screening was performed according to the Preferred Reporting Items for Systematic Review and Meta-Analysis (PRISMA) methodological framework [14]. A comprehensive search strategy was developed to identify relevant articles to be included in our literature review. Using large scientific databases including PubMed, MEDLINE, Embase, and SCOPUS, studies that were published between 2010 and 2020 were collected. Additional search in Google Scholar was conducted to avoid missing any further relevant articles. The search of keywords in various combinations included: “Primary Care” or “Primary Healthcare” or “Primary health care” or “health care delivery” or Primary Medical Care” and “Immigrant” or “Newcomer” and “Patient Experiences” or “Patient satisfaction” or “Patient-Centered Care” or “Quality of Health Care” (Appendix A). Further, to avoid missing any relevant literature, the reference lists of the included articles were searched. The literature search was restricted to the English language.

### 2.2. Inclusion and Exclusion Criteria

The inclusion and exclusion criteria were based on the study objective. The inclusion criteria were primary peer-reviewed articles within the last 10 years that provided information on the immigrant healthcare experience with PHC service in Canada. Studies providing qualitative or quantitative data on patient experiences in the form of discussions or one or more outcome measures were selected for further screening. The exclusion criteria were studies performed outside of Canada, studies that did not cover relevant information about immigrant patient experiences with PHC, studies that focused only on refugee status population, temporary foreign workers, and/or undocumented immigrant, non-English articles, summaries, systematic reviews, abstracts, policy papers, case studies, editorials, and letters.

### 2.3. Data Extraction and Evaluation

All relevant data from finalized articles were extracted by three authors (BBB, SB, and KTB) into a preapproved worksheet. The following information were extracted from each study for collective evaluation: author, year of publication, province/city, study design, participant demographics, study focus, and outcome (experiences/barriers). All authors evaluated the data independently and then reached a consensus on final findings through detailed discussions. Any disagreement between the authors regarding data identification and collection were resolved via thorough discussion and mutual agreement.

### 2.4. Assessment of Quality and Risk of Bias

Two authors (BBB and KTB) independently assessed the quality of included studies by using the Newcastle-Ottawa Scale (NOS) [15], with a rating range from 1 to 10 stars (*). Out of 10 stars, at least six were considered as a high-quality study. The details of the results are presented in Appendix A Table A1. Assessment of bias in selection, comparison, and outcome was conducted as described using the NOS with high quality studies having low risk of bias. Further, publication bias was not quantitively assessed, however, was addressed by using a broad search strategy and a number of scientific and other databases to capture all relevant published and unpublished articles.

## 3. Results

### 3.1. Overview of the Selected Study

**Figure 1:**
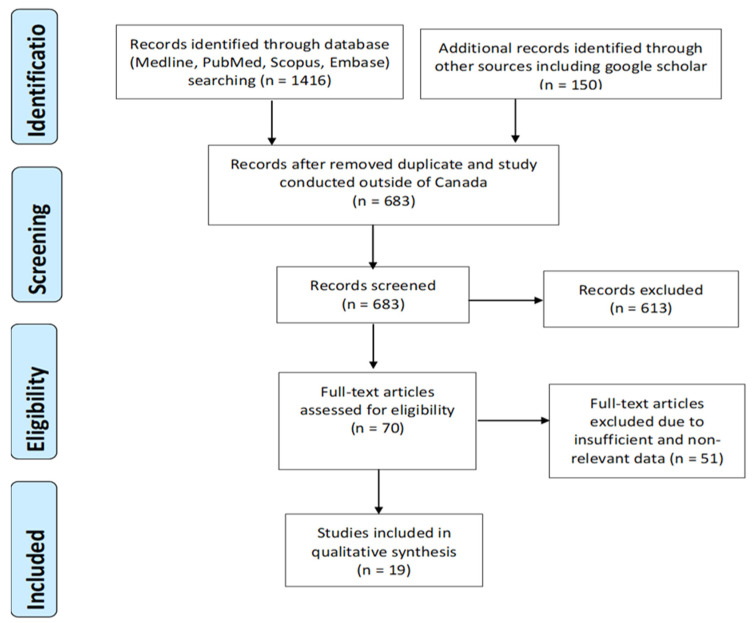
PRISMA flow chart of the literature review and article identification process. PRISMA: Preferred Reporting Items for Systematic Review and Meta-Analysis.

Database and other source (Google Scholar) search resulted in a total of 1566 possibly relevant articles. After removing duplicates and studies outside of the Canada (883), 683 articles were selected for further screening. Based on the inclusion and exclusion criteria described above, 613 studies were removed in this process, and 70 articles were eligible for further full-text screening. Additional 51 articles were eliminated based on lack-of or limited data and information availability, and a total of 19 peer-reviewed papers were finalized for this review (Figure 1).

Of the 19 studies included in the review (Table 1), six were conducted in Ontario [16,17,18,19,20,21], one [18] comparing urban and rural areas, five of the studies [22,23,24,25,26] were done in Alberta and two [23,25] studies conducted in Western Canada. Other study settings included three studies across Canada [27,28,29], two in British Columbia [30,31], one in Manitoba [32], one in Montreal [33], and one in Ottawa [34]. Of the 19 studies, over two-thirds (approx. 68%) of studies employed qualitative methodology, five (n = 5) were quantitative design, and one (n = 1) was mixed method approach (Table 1). Regarding the study population, over one-third of the studies (37%) covered various ethnic groups from South Asia, China, Europe, Latin America, Middle East, Africa, America, Belarus, Colombia, Iraq, Caribbean, Tajikistan. Five of the studies (26%) focused on Chinese and South Asian populations, and two focused only on Chinese populations [29,31]. Other ethnic groups included were from African countries (n = 2), Korean (n = 1), Francophone (n = 1), Iranian (n = 1), Muslim community (n = 1), and Brazilian (n = 1). Over half of the studies (53%) were focused on overall primary healthcare experiences [16,20,21,25,26,28,30,31,32,33]. Four of the studies (21%) were focused on maternity and reproductive health in relation to PHC [23,24,29,34], and other studies focused on cancer [19,27] (n = 2), dental [17,22] (n = 2), and specialist (n = 1) care experiences [18] (Table 2). Among the studies, nearly one-third (32%) focused on women, more than half (63%) on women and men, and one study did not mention the gender of the study population. The range of sample size was from 8 to 7060, and the majority of studies (95%) collected primary data from interviews, focus group discussions, or questionnaires, and one study reported secondary data from the Canadian Community Health Survey (CCHS) [18]. The quality of all selected studies ranged from 7 to 9 in the NOS representing high quality studies and suggesting low risk of biases (Appendix A Table A1).

### 3.2. Summary of the Studies

Overall, the findings from the 19 identified studies (Table 2) revealed four major themes of immigrant patient experiences in accessing and receiving PHC in Canada. These included (1) cultural and linguistic differences—encompassing the relationship between culture and language and how different ethnic groups perceive the world accordingly; (2) socioeconomic challenges—describing social and financial standing of an individual or community and its interplay; (3) health system factors—describing elements related to healthcare organization and policies; (4) patient–provider relationship—representing a fiduciary relationship between a patient and a physician comprising of respect, autonomy, and pledge of highest quality of care. These themes are discussed in further detail below.

#### 3.2.1. Cultural and Linguistic Differences 

The review highlighted several realities related to cultural and linguistic difficulties in accessing and receiving PHC. Physician gender as a cultural barrier in accessing care was prevalent, particularly among South Asian women, as they preferred to have female doctors, especially for reproductive health and physical exams [34]. These preferences not only played a major role in healthcare seeking behaviors among these patients, but also impacted the long-term healthcare decisions for their families and children [34]. Similarly, preference for culturally and linguistically competent care providers was found in the studies conducted by Lee, T.Y. et al., Ngwakongnwi, E. et al., and Wang, L. et al. [20,26,29]. Marshal et al. reported immigrants from South Asian and Chinese backgrounds had an inclination towards evaluating the Canadian healthcare system against that of their own home country, particularly in regard to the availability and choice of provider gender, treatment and diagnostic preference, and access to specialists [30]. Another study by Hulme, J. et al. reported challenges with health screening among Chinese and Bengali immigrant women due to beliefs that symptoms must be present to justify a screening test (Pap, mammography) [19]. Similarly, access to care in these cases was found to be further complicated by preconceived notions of embarrassment and procedural pain [19].

Mental illness or stress was another major cultural barrier observed in immigrant communities as they were considered a matter of disgrace to individuals and the family leading to concealment of problems and underutilization of available resources [19,26]. Further, one study found that immigrants would evade utilizing the services and will place their health at risk if healthcare professionals were not sensitive to their cultural beliefs and values, and if they perceived discrimination and being unvalued [25]. For example, influences from strong family ties to traditional medicine, and a negative perception towards adaptation and acculturation were common themes in many communities [31]. The authors also reported that with such social and cultural stigmas, many immigrants believed that reaching out for their healthcare needs might negatively impact their daily lives and reduce social support from work, family, and community [31].

Language competency was another widely prevalent barrier among immigrant populations with English as their second language. Its impact is not only restricted to the encounter with primary care providers and acute care, but also towards long-term health promotion and disease prevention. Research shows that language is a noticeably big barrier, as identified in a number of studies exploring newcomer experiences, such as difficulties in understanding medical terminology, expressing medical symptoms, missing appointments, feeling disconnected from care, distrust in care providers, services, as well as health information [25,26,29,30]. Immigrants from various communities reported that language barriers lead to anxiety, emotional distress about miscommunication with physicians, and fear of misinterpretation. For example, in one study, language was a common barrier to accessing healthcare among newcomers, immigrants, francophone seniors and the low-German speaking communities, which not only influenced their self-esteem, but led to a feeling of humiliation affecting their continuity of participation in healthcare [35]. As such, immigrant patients strongly desired to have a provider who can communicate with them in their own language and were knowledgeable about their culture [16,20,26,29]. African immigrant and refugee families in particular reported considerable difficulties in communication with the Canadian healthcare system, leading to misunderstandings in diagnosis and treatment plans [32]. Additionally, Harrington et al. found language barriers in accessing healthcare to be common among new Canadians living in rural areas [18]. Here, because of low health/language literacy, lack of linguistically appropriate information, and an inadequate as well as complex process of interpretation services, immigrants reported not only having difficulty understanding medical terminology but also missing appointments, underutilizing available resources, and disgruntlement with the care received [25,26,27]. Limited language proficiency and lack of provider’s training in dealing with immigrant patients led to perceptions of discrimination and exclusion. In one study, immigrant parents reported feelings of loneliness and neglect from primary care providers due to repeated miscommunications and unproductive interactions [22,27]. Overall, differences in culture and language created substantial barriers in accessing and receiving primary healthcare across the majority of immigrant communities. These challenges mainly arose from beliefs and perceptions created by miscommunication and subsequent frustrations [24].

#### 3.2.2. Socioeconomic Challenges 

Canada has a universal healthcare system and individuals’ socioeconomic status should not impact access to physicians and/or the healthcare system, However, evidence suggests that new immigrants nevertheless report financial barriers in accessing primary healthcare [34]. These barriers include struggle in finding a meaningful job, working more than one job, increased working hours, low pay, and an overall struggle to maintain their day-to-day financial needs [23,30,33]. Therefore, financial problem was a big deciding factor for many immigrants in whether or not to seek healthcare at all [16]. Lack of social/family support, lack of insurance coverage, transportation costs, and time were additional barriers in accessing routine care among immigrant parents [22,24]. This was also true in regard to accessing specialized care as well as mental health consultations through a PHC setting [18,28]. Across the majority of immigrant populations, access to primary healthcare was complicated by extra costs (out of pocket) for services such as dental, vision, speech therapy, etc., and thus compromised due to affordability of care for newcomers [20,30,32].

Similarly, social support was reported to have a strong association with accessibility to primary healthcare among the immigrant population in general [22,33,36]. As a new immigrant, patients reported feeling comfortable in accessing care providers when they have family/friends already living in the area [16]. On the other hand, a study among African families stated that due to the lack of social support and networking, they faced challenges in accessing primary healthcare [32]. These families expressed feelings of isolation, neglect, and loneliness voicing emotions such as “you are on your own”. Similarly, a study among Chinese immigrants reported that due to a lack of family, social, or any alternative support system they ended up using expensive private services [29]. These difficulties were further complicated by factors involving adjustment to a new life with changes in employment, schooling, and overall social dynamics [29]. Similarly, difficulties in accessing care were reported due to changes in personal, family, and social responsibilities [18,35]. Cloose et al. reported that immigrant families spend most of their time focusing on basic needs such as food and housing, taking away time for social and healthcare interactions [33].

#### 3.2.3. Health System Structure Factors 

Every country has a different primary healthcare system. In Canada, primary healthcare is the gateway to accessing the healthcare system, meaning patients do not have direct access to specialists or other therapy or diagnostic and therapeutic services. Numerous studies attested to the barriers faced in accessing PHC by newcomers who are coming to a new and unfamiliar healthcare system. Research illustrated that immigrants especially from South Asia, Chinese, the Middle East, as well as francophone migrants expressed limited knowledge about the healthcare system. They often felt overwhelmed, exhausted, fearful, and helpless while interacting with the Canadian healthcare system [25,26,29,30], and even believed that the Canadian healthcare system was less responsive to them [30].

One of the most frequently reported challenges in accessing primary healthcare is the long waiting time to get service and promptness of care [18,20,22,26,29,31]. In a study, families from Africa reported disappointment in the healthcare system, as families expected similar or even better access to care compared to their home country, but were dissatisfied in terms of promptness, availability, and care coordination [32]. Furthermore, Harrington et al. stated that difficulty in accessing specialist care through the PHC system was reported much higher (almost three times) among new immigrants as compared to Canadian-born population resulting in longer wait times and negative perceptions among the immigrant groups [18]. Further, long wait time in diagnosis and treatment, is also reported in a mixed methods study among Korean immigrants, which led them to seek transnational healthcare [20]. Another systemic challenge frequently reported was physical distance to a healthcare establishment. Primary healthcare access was reduced in minority communities and providers with linguistic capabilities were rarely found in geographical approximation of the immigrant groups. These challenges are further escalated when lack of public transportation options and costs for long distance travel are taken into account [16,20,26,29]. Additionally, a study conducted among various immigrants reported that immigrants felt discriminated while accessing PHC. For example, patients reported being refused as a new patient with immigrant status. Participants explained that they were refused to be booked for an appointment from the clinical staff (reception) when their immigration status was known or revealed [21].

Apart from systemic deficits in meeting the immigrant population needs, the majority of these difficulties in dealing with a new healthcare system seems to arise from patient’s lack of information and communication regarding available support systems and resources [24,31]. Lower health literacy in the immigrant population as well as inadequate training of healthcare providers was reported to be amongst the main perceptions for creating these challenges [22,27]. Various studies showed the immigrant narration of difficulties in navigating with the new healthcare system, including challenges in finding healthcare providers and lack of availability of health information in different languages [21,25,26,37].

#### 3.2.4. Patient–Provider Relationship 

Patient–provider relationship is a unique connection between patient and provider that is built via mutual trust, respect, and collaborative understanding of patients’ needs and expectations for improved health outcomes. Openness in communication, accepting patient without any discrimination, involving patient in the decision making process, understanding patient values, beliefs, and culture, and respecting patient’s preferences are some examples of establishing a trusting patient–provider relationship [38].

This review revealed various factors that influenced the patient–provider relationship in immigrant populations. A study within African families reported poor interaction with the provider due to lack of communication regarding medication prescriptions, resulting in negative perception of services and ultimately change in service providers [32]. Moreover, due to healthcare providers’ attitudes and behaviors, immigrants felt intimidated and threatened which led them to stop seeking healthcare services at all. For example, immigrant patients felt that their quality of care was compromised because of the doctor’s inadequate communication and inattentiveness towards their health concern [21]. Similar incidents in other studies have led to perceived discrimination and racism culminating into negative self-perception of health and unmet healthcare needs [21,33]. One study found that doctors showed less empathy towards immigrant patients and the gap in communication led to misunderstandings [25]. Majority of immigrant patients reported lack of shared decision making as a principal component of dissatisfaction in their interaction with healthcare services [16,22,27,39]. For instance, a study of Brazilian immigrant women reported poor perception of behavior/professionalism from their care providers as a result of being excluded from the decision-making process [17]. On the other hand, studies reported having same gender care providers, building trustworthy environments in care, involving patients in the decision-making process, providing complete information fostered good patient experiences and strengthened patient–provider relationships [20,29,34]. Similarly, immigrant patients in another study reported positive perception of the provider if they were receptive towards their views on alternative medicine/therapy (herbal, other supplementary vitamin instead of antibiotic) and discussed these options with their patients [16].

## 4. Discussion

Canada has a publicly funded healthcare system aimed at ensuring equitable care regardless of one’s age, gender, socioeconomic standing, or immigration status [40]. Nevertheless, immigrants face significant barriers to healthcare access in Canada [5,9]. This review presents the broad assessment of the current literature on patient reported experience in accessing and receiving primary healthcare among the immigrant population in Canada. The present review highlighted major themes encompassing these experiences including cultural and linguistic differences, socioeconomic challenges, health system structure factors, and patient–provider relationship in accessing and receiving PHC. Among the four major concerns of immigrant patient experience, cultural and linguistic difference was the most prevalent and challenging aspect that needed to be addressed.

### 4.1. Overcoming Cultural and Linguistic Differences

As Canada has diverse communities that are ethnically heterogenous, it is essential to give special consideration to all cultural dimensions. To provide culturally competent healthcare, care providers need to understand in-depth the cultural distinction of their patient and realize the considerable evidence that show cultural and linguistic barriers are among the major hurdle to proper healthcare access. Canada is not only the country facing this problem, but several European countries and the United States are struggling with similar issues [41,42]. *Physician gender* (preference of having a female doctor) is one of the highest focused cultural barriers among immigrant population especially South Asian, Chinese, and Muslim women, particularly in regard to reproductive health and physical checkup [20,29,30,34]. This review also highlighted *language barriers* as an significant challenge to accessing quality care among the immigrant population [16,19,20,21,22,24,25,26,27,29,30,32,34], which was an equal challenge among primary healthcare providers in delivering quality care to immigrant patients [39,43]. In one study, immigrants experienced medication errors, frequent hospitalization or emergency care visits, as well as dissatisfaction with care due to cultural and linguistic differences [44]. In tackling the cultural and linguistic factors, development and implementation of sensitivity and competency training practices should be promoted across all healthcare professions and facilitators, including clinicians, nurses, allied healthcare professionals, and interpreters. Addition of a more diverse healthcare workforce in terms of race and gender will be a much-needed development in tackling these barriers. Further, to nurture more profound and efficient cross-cultural relationships, incorporating effective health communication training for both care providers and recipients will be beneficial [45].

### 4.2. Facing Socioeconomic and Structural Challenges

Similarly, various socioeconomic challenges in adjusting to a new life, environment, and social structure created major barriers to accessing primary healthcare across immigrant populations. Better understanding of social dynamics in the immigrant population and services aimed at facilitating mutual acculturation can help reduce socioeconomic stressors and improve accessibility to quality care. Furthermore, systemic challenges in the healthcare system create multiple barriers to accessing primary healthcare such as longer wait times, geographical inaccessibility, uncoordinated service, poor response times, and deficient provider to patient ratio, which poses an acute and long-term threat to immigrant health. Integrated and coordinated care, proper transportation facilities, having appropriate patient provider ratio in place are some examples of facilitators that might address the barriers in accessing primary care among immigrant patients. Policies and structural changes geared towards meeting the challenges faced by immigrant communities are needed in this regard for efficient utilization of the PHC services available under the universal healthcare system in Canada.

### 4.3. Improving Patient–Provider Relationship

Development of a positive attitude towards the healthcare system and its providers requires mutual understanding, respect, and acceptance. Forming a respectful and welcoming patient–provider relationship can help overcome the majority of the challenges faced by immigrants, while also prospering the goals of universal healthcare for all. Based on our review, understanding the importance of gender roles specially for female patients and having appropriate staff available; having bilingual service providers and/or interpretation services; paying special attention to new immigrants in understanding their fears and challenges; providing different cultural and language competence training to care providers; most importantly focusing on social and economic contexts of individual patients and approaching those issues early are some important facilitators and strategies to address the significant barriers and to accessing healthcare of immigrants.

The findings of this review can help inform clinicians, healthcare professionals, healthcare administrations, policymakers, and researchers in designing programs specific to the needs and challenges of the immigrant population. Some of the findings from this review include improving interpretation services, increasing number of culturally competent care providers, improving health literacy among immigrant population about the importance of PHC, expanding comprehensive healthcare coverage for dental, eye, and other essential care. The findings of this review also suggest the importance of having patient friendly health information, enhancing social and community supports for newcomers, and promotion of mental health as crucial elements to ensuring long-term success to immigrant health.

This review had several limitations. Included articles were published in English, which could have resulted in missing relevant studies published in a different language, particularly given Canada’s majority English–French bilingualism. Further, we did not include studies that only focused on refugees, temporary foreign workers, and undocumented immigrants, which might have resulted in omission of some relevant population groups. The majority of the studies included for the review were primary data sources and qualitative in terms of study design and analysis. Moreover, only studies focusing on primary healthcare experiences from the patient perspective were included, but not from the provider point of view, who might have similar or/and different challenges while providing care to immigrants. Most of the studies were geographically restricted to Ontario and Alberta which limited inclusion of data from other possibly relevant rural and urban locations. However, in this review, the studies included participants with large ethnic diversity comprising a sizeable composite immigrant population from various social, and cultural backgrounds, that allows the findings to be generalized.

From this literature review, several directions for future research can be pinpointed: qualitative research from care providers and immigrant patients perspective; research focusing on immigrant men (majority of studies were conducted among women); research on availability and utilization of interpretation services and its outcome; research on culturally and linguistically competent care providers and immigrant patient experiences; research on newcomers from diverse groups; health system literacy among newcomers.

## 5. Conclusions

The health characteristics of the immigrant population is a consequence of environmental, economic, genetic, and social-cultural factors of their home country before they migrate to Canada. Post-immigration factors include, a new environment, employment, education, poverty, accessibility and responsiveness of healthcare practitioners and of the healthcare system of Canada [46]. An individual patient’s background and perceptions may influence their interpretation of health and symptoms, coping strategies, healthcare seeking behaviors, decision-making process, preference, and acceptance of treatment within the new healthcare system. Canada is a multicultural country with a diverse ethnocultural landscape. As such, these diversities of patients face a variety of challenges related to accessing the PHC service. In our study, four major themes including culture and linguistic, socioeconomic, healthcare structure, and patient–provider relationship was ubiquitous among the diversity of these immigrant populations. Among these, we identified that culture/linguistic competency, for both patient and physician, played a highly prevalent and crucial role in determining accessibility to the Canadian healthcare system. Similarly, other major themes were also equally important in determining one’s ability and responsiveness in accessing primary care. Together, these factors comprised a common theme in the immigrant healthcare experience and challenges with the PHC system of Canada. It is therefore pertinent that primary care providers play an active role in bridging these gaps for their immigrant patients with strong support from policy level. Development of a positive attitude towards the healthcare system and its providers requires mutual understanding, respect, and acceptance. As immigrants encounter various challenges in accessing and receiving PHC, these challenges need to be addressed both at a systemic level and through efforts from local health organizations to better respond to the needs of immigrant communities. Thus, understanding and respecting diversity in culture, language, experiences, and systems is crucial in reducing health inequalities and improving access to quality care in a respectful and responsive manner.

## Figures and Tables

**Table 1:** Characteristics of the studies included in the review.

Author and Year	Study Population	Sample Size (N)	Length of Stay in Canada (N)	Location	Methodology
Lum, I.D et al. (2016)	*Ethnicity/origin*: Belarus, China, Colombia, Iraq	13: Male = 4, Female = 9	Average 9 years	Ontario	Method: qualitative study Design: cross-sectional, semistructured interview Analysis: thematic analysis
Woodgate, R. L. et al. (2017)	*Ethnicity/origin*: 15 African countries	108: Male = 70, Female = 38	≤6 years	Manitoba	*Method*: qualitative study *Design*: cross-sectional, open-ended interview *Analysis*: thematic analysis
Gulati, S. et al. (2012)	*Ethnicity/origin*: Chinese and South Asian	50: Male = 13, Female = 37	<4 years (7) 4–10 years (18) >10 years (25)	Canada	*Method*: Grounded theory *Design*: cross-sectional, semistructured interview *Analysis*: thematic analysis theory building
Amin, M. et al. (2012)	*Ethnicity/origin*: Ethiopian, Eritrean, and Somali	48 Mothers of 3-years children	<5 years	Edmonton, Alberta	*Method*: qualitative study *Design*: cross-sectional, focus group discussion *Analysis*: thematic analysis health behavior theory
Calvasina, P. et al. (2016)	*Ethnicity/origin*: Brazilian	101: Male = 27, Female = 74	<5 years (80) >5 years (21)	Toronto, Ontario	*Method*: quantitative study *Design*: cross-sectional, self-administered survey *Analysis*: logistic regression
Cloos, P. et al. (2020)	*Ethnicity/origin*: Asia, Caribbean, Europe, Latin America, Middle East, Africa, and United States	806: Male = 283, Female = 495	<5 years (593) ≥5 years (178)	Montreal	*Method*: quantitative study *Design*: cross-sectional, questionnaire *Analysis*: multivariable logistic regression
Harrington, D. et al. (2013)	*Ethnicity/origin*: Immigrants and Born in Canada	7060: Male = 3460, Female = 3600	<10 years (1765) >10 years (5295)	Urban/Rural, Ontario	*Method*: quantitative study *Design*: cross-sectional, CCHS: telephone survey *Analysis*: multivariable logistic regression
Hulme, J et al. (2016)	*Ethnicity/origin*: Chinese and South-Asian (Bangali)	23 Women	<5 years (7) >5 years (10) N/A (6)	Ontario	*Method*: qualitative study *Design*: cross-sectional, semistructured interview and focus group discussion *Analysis*: thematic analysis
Mumtaz, Z et al. (2014)	*Ethnicity/origin*: Asia, Africa, Europe, America	140 women	Since 1996	Alberta, Saskatchewan, Manitoba	*Method*: quantitative study *Design*: cross-sectional, structure CATI *Analysis*: Pearson’s chi square test
Corscadden, L et al. (2018)	*Ethnicity/origin*: Immigrants	18 people	NA	Canada	*Method*: quantitative study *Design*: cross-sectional, survey *Analysis*: logistic regression
Marshall E. G et al. (2010)	*Ethnicity/origin*: Chinese and Punjabi	78: Male = 46, Female = 32	<10 years (52) ≥10 years (26)	British Columbia	*Method*: qualitative study *Design*: cross-sectional, focus group discussion *Analysis*: thematic analysis
Ou, C.H.K et al. (2017)	*Ethnicity/origin*: Chinese	8 Adult	5–19 years (average 14 years)	Vancouver	*Method*: qualitative study *Design*: cross-sectional, structured interview: in-person and telephone *Analysis*: deductive content/themes analysis
George, P et al. (2014)	*Ethnicity/origin*: Muslim- Arab and South Asian countries	22 women	1–25 years	Ottawa	*Method*: qualitative study *Design*: cross-sectional, focus group discussion *Analysis*: thematic analysis
Higginbottom, G. M. et al. (2016)	*Ethnicity/origin*: Sudan, Philippines, China, Columbia, Tajikistan, India, Mauritania, Pakistan Eritrea	34 women	NA	Rural/Urban, Alberta	Method: qualitative study Design: ethnographic research, semistructured interview Analysis: Roper and Shapira’s framework analysis
Lee, T.Y. et al. (2014)	*Ethnicity/origin*: Chinese (China, Hong Kong, Taiwan)	15 women	<10 years	Canada	*Method*: qualitative-descriptive phenomenology *Design*: cross-sectional, semistructured interview *Analysis*: thematic analysis
Dastjerdi, M. et al. (2012)	*Ethnicity/origin*: Iranian community)	17: Male = 6, Female = 11	2–15 years	Western Canada	*Method*: qualitative study *Design*: constructive grounded theory interview *Analysis*: thematic analysis theory building
Ngwakongnwi E. et al. (2012)	*Ethnicity/origin*: Francophone Immigrant	26: Male = 11, Female = 5	<10 years (16) >10 years (6)	Calgary, Alberta	*Method*: qualitative descriptive study *Design*: cross-sectional, semistructured telephone interview *Analysis*: thematic analysis theory building
Wang, L. et al. (2015)	*Ethnicity/origin*: Korean community	351: Male = 173, Female = 178	<5 years (17) 5–9 years (6) 10–19 years (14) >20 years (16)	Toronto	*Method*: Mixed methods *Design*: cross-sectional, CCHS survey and focus group discussion *Analysis*: Z-test, thematic analysis
Pollock, Grace et al. (2012)	*Ethnicity/origin*: Middle Eastern, African, Latin American, South Asian, eastern European, Caribbean	26: Male = 7, Female = 19	NA	Ontario	*Method*: qualitative study *Design*: cross-sectional, semistructured interview *Analysis*: thematic analysis

Note: CATI: Computer-Assisted Telephone Interviewing; CCHS: Canadian Community Health Survey; NA: not available.

**Table 2:** Findings of the studies included in the review.

Author and Year	Study Focus	Patient Experiences/Barriers Mentioned
Lum, I.D et al. (2016)	Examining the experiences of immigrants living in a small urban center—primary healthcare system	Factors impacting access to primary care: 1. Lack of social contacts 2. Lack of universal healthcare coverage during their initial arrival 3. Language as a barrier 4. Treatment preferences 5. Geographic distance to primary care
Woodgate, R. L. et al. (2017)	Examining the experiences of access to PHC by African immigrant and refugee families	Major barriers to primary care services: 1. Expectation not quite met: accessibility, promptness of services, availability, affordability, and acceptable of services 2. Facing a new life in unfamiliar environment 3. Linguistic and cultural differences, lack of social support/network
Gulati, S. et al. (2012)	Exploring the role of communication and language in the healthcare experiences of immigrant parents of children with cancer living	Barriers to care: 1. Language/communication challenges influenced parents’ role in caring child 2. Health literacy—difficulty to understand medical terminology, inadequate interpretation services, occasionally missed resources, reported limited availability of linguistically and culturally appropriate information 3. Lack of social integration in the healthcare process and competence
Amin, M. et al. (2012)	Identifying psychosocial barriers to providing and obtaining preventive dental care for preschool children among African recent immigrants	Barriers were associated with: 1. Home-based prevention: health beliefs, knowledge, oral health approach, skills 2. Perceived role of caregivers and dentists 3. Role of parental knowledge in access to professional care and preventive services, attitudes toward dentists and services, English skills, and external constraints concerned dental insurance, social support, time, and transportation.
Calvasina, P. et al. (2016)	Investigating the association between oral (dental) health literacy (OHL) and participation in oral healthcare among Brazilian immigrants	83.1% had adequate OHL; low OHL and access of care was associated with: 1. Not visiting a dentist or having a dentist as the primary source of information 2. Not participating in shared dental treatment decision making: language barrier 3. Low average annual income of household
Cloos, P. et al. (2020)	Examining the social determinants of self-perceived health of migrants with precarious status (MPS)	Almost half 44.8% perceived their health as negative. Barriers were reported as: 1. Having no diploma/primary/secondary education 2. Unmet needs due to low family income 3. No financial backup or assistance resources 4. Perception of racism 5. Feeling of psychological distress 6. Unmet healthcare needs; having one or more health issue in past 12 months
Harrington, D. et al. (2013)	Barriers in accessing to specialty care for all populations including subgroup of immigrant populations	1. Newcomers (69.2%) and longer-term immigrants (72.1%) were more likely to report difficulties with wait times compared to Canadian-born (64.3%). 2. 14.2% newcomers experienced difficulties: transportation, cost, or language 3. Newcomers reported due to personal or family responsibilities they experience difficulties (23.3%) compared to Canadian-born (9.8%) and longer-term immigrant respondents (10.5%)
Hulme, J et al. (2016)	Exploring perception of Chinese and South-Asian immigrants regarding breast and cervical cancer screening	Major themes reported were: 1. Risk perception and concepts of preventative health and screening—"painful or traumatic encounters" 2. Health system engagement and the embedded experience with screening:Female provider vital 3. Fear of cancer and procedural pain 4. Self-efficacy, obligation, willingness to be screened 5. Newcomer barriers and competing priorities: new healthcare system, language, transportation, childcare, work, limited social network, and cultural
Mumtaz, Z et al. (2014)	Exploring newcomer women’s experiences in Canada regarding pregnancy, delivery, and postpartum care	1. Financial—newcomers were more likely to be university graduates but had lower incomes than Canadian-born women. 2. Information—no differences found-newcomer’s ability to access acceptable prenatal care, but fewer received information: emotional/physical changes during pregnancy
Corscadden, L et al. (2018)	Assessing the factors associated with multiple barriers in accessing and care-seeking process in different healthcare systems	Barriers to accessing primary care clinic: 1. After-hours access very difficult 2. Over five days to get appointment; no timely response to call 3. Cost for medicines, clinic visit 4. Care not coordinated 5. GP did not spend enough time/unclear explanation.
Marshall E. G et al. (2010)	Conceptualizing unmet healthcare needs and primary healthcare experiences among Chinese- and Punjabi-speaking immigrants	Experiences and barriers to accessing care: 1. Costly dental and speech therapy (trade-off between service and paying out-of-pocket) 2. Lack of choice in the gender of a provider 3. Lack of primary care provider accepting new patient, speaking patient’s language 4. Lack of health system literacy: limited knowledge; not enough information provided by service providers; less responsive healthcare system 5. Language is a noticeably big barrier to understanding medical terminology, information about health
Ou, C.H.K et al. (2017)	Examining the health beliefs, health behaviors, primary care access, and perceived unmet healthcare needs of Chinese young adults	Barriers experienced: 1. Inaccessibility to primary care provider and preventive services. 2. Influence of cultural factors such as strong family ties, filial piety, and practice of Traditional Chinese Medicine on healthcare behaviors and access 3. Long wait for specialist 4. Low literacy about healthcare system (dental)
George, P et al. (2014)	Understanding health behaviors of the minority population and identifying barriers to accessing reproductive health	Health seeking behavior barriers identified: 1. Gender of physician for reproductive health 2. Preference of family physician from same ethnic and cultural background 3. Language barrier while communicating with care providers
Higginbottom, G. M. et al. (2016)	Understanding immigrant women’s experiences in maternity healthcare and devising potential intervention that might improve the experiences and outcomes	Barriers reported in accessing care: 1. Communication difficulties 2. Lack of Information 3. Lack of social support 4. Cultural belief 5. Inadequate healthcare services 6. Cost of medicine/services
Lee, T.Y. et al. (2014)	Exploring immigrant Chinese women’s experiences in accessing maternity care, the utilization of maternity health services, and the obstacles they perceived	Patient preference/experiences: 1. Preference of having linguistically and culturally competent healthcare providers 2. Dealing with different healthcare system—felt complex 3. Having information in different language felt convenient, but insufficient 4. Satisfied with Canadian Healthcare System, but long wait time long transportation (distance) 5. Felt lacking alternative support
Dastjerdi, M. et al. (2012)	Exploring and understanding the experience of Iranian immigrants who accessed Canadian healthcare services	Barrier facing in accessing healthcare: 1. Language barriers (missed appointment, could not trust healthcare providers and services) 2. Financial barriers 3. Cultural difference: felt discrimination and unvalued: *help was not sought and waiting to be asked* 4. Felt marginalized, humiliation—interpretation services process 5. New healthcare system: felt overwhelmed, exhausted, and burned out 6. Hard to understand health information—available only in English
Ngwakongnwi E. et al. (2012)	Examining healthcare access experiences of immigrants and nonimmigrants, French speakers in a mainly English-speaking province of Canada	Barrier reported in accessing care: 1. Difficulty finding family doctor 2. Language barrier: difficulty to explain, emotional distress prior to visit doctor, feelings disconnected, delay in seeking care 3. Complexity of language interpreter service 4. Preference of having culturally and linguistically competent providers 5. Knowledge of healthcare system, transportation barriers, and cost of drugs for recent immigrant
Wang, L. et al. (2015)	Capturing health status and experiences in accessing local and transnational healthcare among South Korean immigrants	Preference and barriers expressed: 1. Social cultural and language barriers: preference of Korean-speaking physician, not understanding medical terms, hard to express medical symptoms 2. Lack of social network and support felt left behind. 3. Geographic barriers: long distance to see doctor 4. Economic barrier: lack of extended health insurance for drugs, dental, vision, and other essential care, using traditional Chinese medicine 5. Seeking transnational healthcare: accessing healthcare from Korea physically/online/telephone 6. Waiting time: nearly half participants expressed long wait for diagnosis and treatment 7. Overall, favorable healthcare system, but expected to have comprehensive annual health checkup
Pollock, Grace et al. (2012)	Capturing the perceptions of discrimination from a service user perspective in five small and medium-sized Ontario cities	Participant-reported experiences: 1. Refusal to provide healthcare as an immigrant 2. Staff acting as a gatekeeper: refused patient from the reception 3. Communication/language barriers and culturally insensitivity 4. Perceived discrimination: compromised the quality of care, doctor’s inattentiveness about health concerns

## Appendix A

Appendix A. Literature search performed using various online scientific databases between July 5–10.

Literatures found from publications between 2010 upto 10 July 2020 in a variety of databases Medline (N = 508), Embase (N = 121), PubMed (N = 680), Scopus (107), Google Scholar (N = 150) resulted in a total of 1566 hits. After proper screening process, 19 articles were selected for final review. Overall screening process and results can be found in Figure 1. (PRISMA flowsheet).

**Medline search 10 July 2020.**

**No.**	**Search terms/limits**	**Result**
1	Primary Care.mp. or exp Primary Health Care/	229,630
2	exp Primary Health Care/ or exp Patient Satisfaction/ or exp "Quality of Health Care"/ or Patient Experience.mp. or exp Patient-Centered Care/	7,026,134
3	1 and 2	197,116
4	Immigrant.mp. or exp "Emigrants and Immigrants"/	21,250
5	3 and 4	727
6	limit 5 to english language	659
7	limit 6 to yr="2010 -Current"	523
8	limit 7 to journal article	508

**Embase search 10 July 2020.**

**No.**	**Search terms/limits**	**Result**
1	primary health care/ or exp health care delivery/	3,300,025
2	Experiences.mp.	237,561
3	1 and 2	45,464
4	exp immigrant/	16,428
5	3 and 4	171
6	limit 5 to english language	169
7	limit 6 to yr=”2010 -Current”	121

**PubMed, Scopus & Google Scholar search between July 5-July 10, 2020:**

PubMed search using [((((((Primary Care[Title]) OR (Primary Healthcare[Title])) AND (Immigrant[Title])) OR (Immigrant population[Title])) OR (Newcomer[Title])) AND (Patient Experiences[Title])) OR (Immigrant Patient Experiences[Title]) with language (English), timeframe (2010-202), Full text Journal Article restriction yielded 680 results.

Scopus search using (TITLE-ABS-KEY(Primary Care) OR TITLE-ABS-KEY(Primary Healthcare) OR TITLE-ABS-KEY(Primary Medical Care) AND TITLE-ABS-KEY(Immigrant) AND TITLE-ABS-KEY(Experience)) AND PUBYEAR > 2009 AND (LIMIT-TO (DOCTYPE,"ar")) AND (LIMIT-TO (LANGUAGE,"English")) AND (LIMIT-TO (SRCTYPE,"j")) yielded 107 results.

Google Scholar search using key words ["Primary Care" "Immigrant population" "Patient experience"] with language (English) and timeframe (2010-2020) restrictions yielded 150 results.

**Table A1:** NEWCASTLE-OTTAWA quality assessment scale for cross sectional studies.

Author(s) and Year	Selection	Comparability	Exposure/Outcome	Total Score
Lum, I.D et al., 2016	***	**	***	8
Woodgate, R. L. et al., 2017	***	**	***	8
Gulati, S. et al., 2011	****	**	***	9
Amin, M. et al., 2012	***	*	***	7
Calvasina, P. et al., 2016	***	**	***	8
Cloos, P. et al., 2020	***	**	***	8
Harrington, D. et al., 2013	***	**	***	8
Hulme, J et al., 2016	***	**	***	8
Mumtaz, Z et al., 2014	***	*	***	7
Corscadden, L et al., 2018	***	*	***	7
Marshall E. G et al., 2010	***	**	***	8
Ou, C.H.K et al., 2017	***	*	***	7
George, P et al., 2014	***	*	***	7
Higginbottom, G. M. et al., 2016	****	**	***	9
Lee, T.Y. et al., 2014	***	**	***	8
Dastjerdi, M. et al., 2012	***	**	***	8
Ngwakongnwi E. et al., 2012	****	**	***	9
Wang, L. et al., 2015	***	*	***	7
Pollock, Grace et al., 2012	***	*	***	7
